# Association between leukocyte telomere length and the risk of pancreatic cancer: Findings from a prospective study

**DOI:** 10.1371/journal.pone.0221697

**Published:** 2019-08-29

**Authors:** Hung N. Luu, Joyce Y. Huang, Renwei Wang, Jennifer Adams-Haduch, Aizhen Jin, Woon-Puay Koh, Jian-Min Yuan

**Affiliations:** 1 Division of Cancer Control and Population Sciences, UPMC Hillman Cancer Center, University of Pittsburgh, Pittsburgh, PA, United States of America; 2 Department of Epidemiology, Graduate School of Public Health, University of Pittsburgh, Pittsburgh PA, United States of America; 3 Health Services and Systems Research, Duke-NUS Medical School Singapore, Singapore; 4 Saw Swee Hock School of Public Health, National University of Singapore, Singapore; CUNY School of Public Health, UNITED STATES

## Abstract

**Introduction:**

Telomeres and telomerase play important role in maintaining chromosome integrity and genomic stability. Recent epidemiologic data showed inconsistent findings which suggested that both short and long leukocyte telomeres could be associated with increased risk of pancreatic cancer. We prospectively examined the association between telomere length and pancreatic cancer risk in a population-based cohort study.

**Methods:**

The Singapore Chinese Health Study recruited 63,257 Chinese aged 45 to 74 years from 1993 to 1998 in Singapore. Relative telomere length in peripheral blood leukocytes was quantified using a validated monochrome multiplex quantitative polymerase chain reaction method in 26,540 participants, including 116 participants who later developed pancreatic cancer after an average of 13 years of follow-up. Cox proportional hazard regression method was used to calculate hazard ratio (HR) and its 95% confidence interval (CI) of pancreatic cancer risk associated with telomere length, with adjustment for confounding factors.

**Results:**

Longer telomeres were significantly associated with higher risk of pancreatic cancer (*P*_trend_ = 0.02). Compared with lowest quartile, subjects with highest quartile of telomere length had an HR of 2.18 (95% CI: 1.25–3.80) for developing pancreatic cancer. In stratified analysis, this association remained among pancreatic adenocarcinoma patients but not among pancreatic non-adenocarcinoma patients. In continuous scale, the HRs and 95% CIs were 3.08 (1.17–8.11) for adenocarcinoma patients and 1.47 (0.43–5.06) for non-adenocarcinoma patients. The HRs and 95% CIs of the highest quartile of telomere length, compared with the lowest quartile, for adenocarcinoma and non-adenocarcinoma were 2.50 (1.22–5.13) and 1.63 (0.66–4.03), respectively. The length of follow-up from the collection of blood for the measurement of telomere length to the diagnosis of cancer (median = 8.0, range: from 5.0 months to 16.2 years) had no significant impact on the association between telomere length and pancreatic cancer risk.

**Conclusions:**

The present study demonstrates that longer telomeres are associated with increased risk of overall pancreatic cancer.

## Introduction

Although pancreatic cancer may be uncommon, it is a highly lethal malignancy. Worldwide, pancreatic cancer ranks 7^th^ in cancer mortality with approximately 432,250 deaths out of 459,000 new cases a year [1]. In the U.S., it ranks 4^th^ in cancer mortality in both sexes [2]. Compared to other more common cancers, pancreatic cancer has relatively short survival time and the low 5-year survival rate after cancer diagnosis [2] as most cases are diagnosed at an advanced stage. Moreover, while incidence and mortality rates of other cancers have been declining in the past four decades, the incidence rate of pancreatic cancer has been increasing by 1.5% per year [2,3], and the survival rate of pancreatic cancer patients remained constant over the same period [2,4]. Established risk factors for pancreatic cancer include chronic pancreatitis, obesity, type 2 diabetes, and tobacco smoking [5]. Collectively, these risk factors are attributable to less than half of pancreatic cancer burden in the U.S [5]. Hence, there is an urgent need to identify the underlying risk factors of pancreatic cancer to understand the process of carcinogenesis for better prevention and control of pancreatic cancer.

Telomeres, which are tandem DNA repeats at the ends of chromosomes, and telomerase, an enzyme that maintains telomere length, play important role in genomic stability as they protect chromosome ends from degradation, fusion and irregular recombination [6,7]. Human telomeres are approximately 10–15 kb and shorten approximately 30–200 bp after each cycle of cell division [8]. The rate of telomere shortening across individuals depends on both genetic and environmental factors (i.e., smoking, diabetes, obesity or physical activity) [9,10]. In normal circumstances, inactivity of telomerase and the incomplete replication of linear DNA molecules at the end of each chromosome result in telomere shortening, leading to cell senescence and triggering the programmed cell death (apoptosis) [6]. Cell senescence and apoptosis can prevent malignant transformation of damaged cells, thus reducing the likelihood of cancer development. On the other hand, under abnormal circumstances, cycles of cell division do not cause telomere shortening, may be through the activation of telomerase or other unknown mechanisms, resulting in evading cell senescence and apoptosis, considering a hallmark of cancer [11]. Consequently, individuals with longer telomeres relative to their counterparts at the same chronological age may have a higher chance of developing cancer.

Previous epidemiologic studies showed mixed findings that both short [12–14] and long leukocyte [12,14–16] telomere lengths were associated with increased risk of pancreatic cancer. Possible underlying factors accounting for these inconsistent results might be variations in study design, characteristics of study populations or follow-up duration, time between blood collection and the diagnosis date of pancreatic cancer, methods used to measure telomere length in different laboratories and other confounding factors that may impact on telomere length [17]. The Mendelian randomization approach, which usually avoids the potential confounding effect of environmental exposure on the relation between phenotypically measured telomere length and risk of pancreatic cancer, has shown that genetically predicted telomere length is not associated with pancreatic cancer risk [18]. They, however, observed that one short telomere-related allele (*rs10936599*, T) was associated with decreased risk of pancreatic cancer but the other short telomere-related allele (*rs2736100*, A) was associated with increased risk of pancreatic cancer.

These conflicting results from previous epidemiologic studies prompted us to perform the present analysis to examine the association between leukocyte telomere length and risk of pancreatic cancer in a prospective cohort study in Singapore.

## Methods

### Study population

The current analysis was based on the data from the Singapore Chinese Health Study, a population-based prospective cohort study established between April 1993 and December 1998 by the recruitment of middle-age and elderly Chinese living in government-built housing estates, where 86% of the Singapore population resided in during the period of recruitment. The participants belonged to either the Hokkien dialect group who originated from the Fujian province, or the Cantonese dialect group who originated from the Guangdong province in southern China. Detailed information on designs and methods has been described elsewhere [19]. Briefly, at baseline, participants were interviewed at their homes by trained interviewers, using a structured questionnaire to collect information on demographics, body weight and height, lifetime use of tobacco, current physical activity, menstrual/reproductive history (women only), occupational exposure, medical history, and family history of cancer. Body mass index (BMI) was calculated as the weight in kilograms divided by height in meters squared. All study participants provided written informed consent. The present study was approved by the Institutional Review Boards of the National University of Singapore and the University of Pittsburgh.

Blood and urine samples were initially collected from a 3% random sample of cohort participants in 1994–1999. Between July 1999 and December 2003, all surviving cohort subjects were re-contacted for a telephone interview to update information on alcohol use, tobacco use, medical history, current physical activity, and body weight. At the telephone interviews, participants were asked to donate blood. We began blood and urine collection from all consented surviving cohort participants at the beginning of year 2000. For those declining our request for blood donation, mouthwash samples were collected instead.

Of all the subjects that we re-contacted successfully, 28,346 subjects (approximately 57%) consented to donating blood for research. The study participants who provided blood samples were younger than those who did not (mean ± standard deviation-SD: 60.9 ± 7.7 versus 62.4 ± 8.2 years of age), more educated (33.6% versus 25.1% having secondary or higher education), more likely to be men (45.5% versus 39.1%), and had slightly higher prevalence of smoking (32% versus 30.2% ever smokers) and regular alcohol consumption (18.3% versus 14.8% weekly or daily consumers of alcohol).

### Dietary assessment

Dietary assessment in SCHS used semi-quantitative food frequency questionnaires (FFQs) that was validated against a series of 24-hour dietary recall (24-HDR) interviews [20] and selected biomarker studies on random subsets of cohort participants [21,22]. The SCHS FFQ contained 165 food items and food groups commonly consumed in Singapore. Study participants were asked how frequently (in 8 categories: ranging from “never or hardly ever” to “two or more times a day”) they consumed the food or food group and followed by a question on the amount of food consumed, using photographs to choose from three portion sizes (small, medium, large). Average daily intake of approximately 100 nutrients and non-nutrient compounds was computed for each study participant using the Singapore Food Composition Database [20]. The reproducibility and validity of the SCHS FFQ were evaluated in 1,022 individuals with two 24-hour recalls, one weekday and one weekend during 12 months. The correlation coefficients for the majority of calorie-adjusted nutrients ranged from 0.50 to 0.75 [20].

For each of the 4 types of alcoholic beverages (beer, wine, western hard liquor, Chinese hard liquor), participants were asked to choose from eight frequency categories: never or hardly, once a month, 2–3 times a month, once a week, 2–3 times a week, 4–6 times a week, once a day, and 2 or more times a day. Consumers were then asked to choose from four defined portion sizes. For beer, the portion sizes were 1 small bottle (375 ml) or less, 2 small bottles or 1 large bottle (750 ml), 2 large bottles, and 3 large bottles or more. For wine, the portion sizes were 1 glass (118 ml) or less, 2, 3, and 4 glass or more. For Chinese or western hard liquor, the portion sizes were 1 shot (30 ml) or less, 2, 3, and 4 shots or more. One drink was defined as 375 ml of beer (13.6 g of ethanol), 118 ml of wine (11.7 g of ethanol), or 30 ml of western or Chinese hard liquor (10.9 g of ethanol).

### Assessment of pancreatic cancer cases

Incident pancreatic cancer cases and all deaths within the cohort was identified, using the International Classification of Diseases-Oncology, 2^nd^ Edition code C25 via linkage of all cohort participants with the database of the nationwide Singapore Cancer Registry and the Birth and Death Registry that have complete records of incident cancer and death cases in Singapore, respectively [23]. All histologic types of pancreatic cancer were included in the current analysis, including neoplasm malignant (n = 5), carcinoma (n = 4), pleomorphic carcinoma (n = 1), adenocarcinoma (n = 49), mucinous adenocarcinoma (n = 3), infiltrating duct adenocarcinoma (n = 13), carcinoid tumor malignant (n = 1), neuroendocrine carcinoma (n = 1), intraductal papillary-mucinous carcinoma (n = 2), as well as 38 pancreatic cancer cases with unknown histology. Of the total 116 cases, 65 were adenocarcinoma and the remaining 51 were other/unknown histology of pancreatic cancer. The ascertainment of cancer incidence and deaths among all study participants were virtually complete as to the time of this study, only 56 (<0.1%) of the entire cohort participants were known to be lost to follow-up due to migration out of Singapore or for other reasons.

### Measurements of leukocyte telomere length

Genomic DNA was extracted from peripheral blood using QIAamp 96 DNA Blood kits (Qiagen, Valencia, CA) according to the manufacturer’s protocol. Leukocyte telomere length was measured using a validated monochrome multiplex qPCR method, as described elsewhere [24]. Briefly, this method measures the relative average telomere length in genomic DNA by determining the ratio of telomere repeat copy number (T) to single (albumin) gene copy number (S) in experimental samples relative to a reference sample (the T/S ratio). The DNA sample for the standard curve was composed of an equimolar pool of 77 samples selected from participants of the Singapore Chinese Health Study who were identified in a prior study; the telomere length values of all the 77 samples were within 10% of the population mean. This pooled DNA sample was run on all qPCR plates: 8 replicates for each of four concentrations (4, 0.8, 0.16, and 0.032 ng/μl). Thermal cycling was carried out on an Applied Biosystem 7900 HT instrument, using PCR cycling conditions as described previously [24]. Real-time PCR cycle thresholds, determined independently for the albumin gene (ALB) and telomere (TEL) amplification traces for all wells (experimental and standard DNA samples), were used to calculate telomere length [24] with the 384-well plate-based normalization of telomere length, which was more robust than the overall standard-curve based normalization. All experimental DNA samples were assayed in duplicate, and the average value of the two replicates was used for final analysis for each subject. The mean intra-assay coefficient of variation, as a measure of reproducibility for telomere length, was 3.5% over all technical sample duplicates.

### Statistical analysis

The relative average telomere length (i.e., the T/S ratio) for each individual was included in the final statistical analysis. After excluding 1,585 participants with a history of cancer at the time of blood collection and an additional 221 subjects with unavailable telomere length measurement due to assay problems, the current analysis included 26,540 subjects. As of December 31, 2016, with an average follow-up of 12.8 years after their donation of blood sample, 116 among them had developed pancreatic cancer. For each study participant, person-years at risk were counted from the date of blood draw to the date of pancreatic cancer diagnosis, death, migration out of Singapore, or December 31, 2016, whichever occurred first.

Means and standard deviations (SDs) were calculated for continuous variables while counts and proportions were calculated for categorical variables. We used the *t*-test to compare differences for continuous variables and the χ^2^ test to compare differences for categorical variables between pancreatic cancer cases and the rest of the cohort participants, as well as across quartiles of telomere length.

Cox proportional hazard regression method was performed to calculate hazard ratios (HRs) and their corresponding 95% confidence intervals (CIs) for developing pancreatic cancer associated with higher quartiles compared with the lowest quartile. We tested for linear trend by treating the telomere length quartiles as an ordinal variable in the Cox proportional hazard model. We did not find violation of the proportional hazards assumption in our dataset when we examined this assumption using time-varying covariates (i.e., an interaction between leukocyte telomere length and the event time in log) in the Cox models (*P* = 0.876).

All Cox proportional hazard regression models included measures of smoking history–number of cigarettes smoked per day (never smokers, 1–12, 13–22, or 23+), number of years of smoking (never smokers, 1–19, 20–39, or 40+), and number of years since last smoked for quitters (current smokers, <1, 1–4, 5–19, 20+ years since last smoked, or never smokers). Other potential confounders included in the multivariate Cox proportional hazards models were age, sex, dialect group (Hokkien or Cantonese), level of education (no formal education, primary school, secondary or higher education), body mass index (<20, 20 to <24, 24 to <28, or ≥28 kg/m^2^), and alcohol consumption (non-drinkers, 1 to <7, or ≥7 drinks per week), history of diabetes (no or yes), and physical activity (no or yes). The weekly physical activity was defined as any moderate or vigorous activity, or strenuous sports lasting at least 30 minutes. In the current analysis, all information of participant characteristics, except education, were from follow-up 1 interview, which were closer to the sample collection date. The BMI categories was grouped based on the recommendation for Asians by the World Health Organization (WHO) [25]. These covariates were selected based on findings from our prior study [26] and others [5,27]. Specifically, in the previous study [26], we found that shorter leukocyte telomeres were associated with older age, male gender, lower level of education, ever smoking, daily drinkers of alcoholic beverages, and less physical activity. These factors were also associated with higher risk of pancreatic cancer. Therefore, these risk factors were included as covariates in the multivariable Cox regression models.

We also performed stratified analysis and sensitivity analyses by examining the robustness of the association between leukocyte telomere length and pancreatic cancer risk. Major risk factors for sub-group analyses were: smoking status (ever vs. never smoked), sex (male vs. females), BMI status (<25 vs. ≥25), and alcohol consumption (non-drinkers vs. drinkers). For sensitivity analysis, we performed a stratified analysis using median duration from blood draw to the date of diagnosis of pancreatic cancer (<8 years vs. ≥8 years). We conducted additional analysis after excluding cases and person-years observed within the first 3 years after blood draw. All statistical analyses were conducted using SAS, version 9.4 (SAS Institute Inc., Cary, NC). All *P* values presented are two-sided, and *P* < 0.05 was considered statistically significant.

## Results

The mean (±SD) age at the cancer diagnosis of the 116 pancreatic cancer cases identified in this cohort was 74.1 (±8.3) years. The median time interval between blood collection and pancreatic cancer diagnosis was 8.0 years (from 5.0 months to 16.2 years).

**Table 1** compares the baseline characteristics of pancreatic cancer patients with the remaining participants of the cohort. Cases were older than non-cases. The differences in distributions of other demographic and lifestyles between cases and non-cases were not statistically significant (all *P’*s >0.05).

**Table 1:** Distributions of characteristics among study participants. The Singapore Chinese Healthy Study, 1993–2016.

Characteristics	Cases (n = 116) n, %	Non-cases (n = 26,422) n, %	P-value
Mean age (±SD), years^b^	66.24±7.86	62.80±7.64	<0.0001
Gender (%)^a^			
Male	60 (51.72)	12,173 (46.07)	0.22
Female	56 (48.28)	14,249 (53.93)	
Dialect^a^			
Cantonese	61 (52.59)	13,405 (50.73)	0.69
Hokkien	55 (47.41)	13,017 (49.27)	
Highest level of education (%)^a^			
No formal education	24 (20.69)	5,501 (20.82)	0.65
Primary school	57 (49.14)	11,974 (45.32)	
Secondary school or higher	35 (30.17)	8,947 (33.86)	
Mean body mass index (±SD)^b^, Kg/m^2^	23.20±3.78	23.25±3.50	0.95
Smoking status (%)^b^			
Never smoker	72 (62.07)	17,949 (67.93)	0.23
Former smoker	25 (21.55)	4,181 (15.83)	
Current smoker	19 (16.38)	4,292 (16.24)	
Alcohol consumption (%)^b^			
Non-drinkers	92 (79.31)	21,484 (81.31)	0.85
1 to <7 drinks/week	18 (15.52)	3,753 (14.20)	
≥7 drinks/week	6 (5.17)	1,185 (4.49)	
Diabetes (%)^b^			
No	95 (81.90)	22,694 (85.90)	0.22
Yes	21 (18.10)	3,728 (14.10)	
Any weekly physical activity*^a^ (%)			
No	76 (65.52)	16,985 (64.27)	0.78
Yes	40 (34.48)	9,437 (35.73)	
Mean time from blood collection to cancer diagnosis (±SD)	8.88±4.23	-	

*The weekly physical activity was defined as any moderate or vigorous activity, or strenuous sports lasting at least 30 minutes

^a^Variables reported at baseline

^b^Variables reported at the follow-up 1.

**Table 2** shows selected characteristics of participants by relative average telomere length (i.e., T/S ratio). Compared to those in the lowest quartile for telomere length, those in the highest quartile were expectantly about 5 years younger in average at the time of blood sampling. They also had slightly higher BMI and were more likely to be women or engage in weekly physical exercise while were less likely to smoke, drink alcohol daily or report a history of diabetes (*P*<0.02).

**Table 2:** Distributions of Baseline characteristics among study participants by relative telomere length. The Singapore Chinese Health Study, 1993–2016.

		Relative Average Telomere Length (T/S ratio)* in Quartile [Median] and Range				
	Total # Subjects	1^st^ Quartile 0.78 (0.19–0.87)	2^nd^ Quartile 0.93 (0.87–0.99)	3^rd^ Quartile 1.07 (1.00–1.15)	4^th^ Quartile 1.27 (1.15–3.24)	*P-*value
Number of subjects	26,540	6,635	6,635	6,635	6,635	
Mean age (±SD), years^b^	26,540	65.60±7.80	63.37±7.53	61.99±7.32	60.30±6.90	<0.0001
Gender (%)^a^						
Male	12,234	3,637 (54.82)	3,212 (48.41)	2,852 (42.98)	2,533 (38.18)	<0.0001
Female	14,306	2,998 (45.18)	3,423 (51.59)	3,783 (57.02)	4,102 (61.82)	
Dialect^a^						
Cantonese	13,468	3,308 (49.86)	3,324 (50.10)	3,347 (50.44)	3,489 (52.58)	0.006
Hokkien	13,072	3,327 (50.14)	3,311 (49.90)	3,288 (49.56)	3,146 (47.42)	
Highest level of education (%)^a^						
No formal education	5.526	1,469 (22.14)	1,374 (20.71)	1,383 (20.84)	1,300 (19.59)	<0.0001
Primary school	12,032	3,136 (47.26)	2,949 (44.45)	3,012 (45.40)	2,935 (44.24)	
Secondary school or higher	8,982	2,030 (30.60)	2,312 (34.85)	2,240 (33.76)	2,400 (36.17)	
Mean body mass index (±SD)^b^, Kg/m^2^	26,540	23.13±3.49	23.26±3.54	23.32±3.49	23.30±3.51	0.01
Smoking status (%)^b^						
Never smoker	18,021	4,027 (60.69)	4,402 (66.35)	4,685 (70.61)	4,907 (73.96)	<0.0001
Former smoker	4,207	1,378 (20.77)	1,103 (16.62)	903 (13.61)	823 (12.40)	
Current smoker	4,312	1,230 (18.54)	1,130 (17.03)	1,047 (15.78)	905 (13.64)	
Mean cigarettes/day (±SD)	8,519	17.42±13.03	16.75±13.05	16.69±12.65	16.55±12.60	0.09
Mean years of smoking (±SD)	8,519	36.15±14.62	34.18±14.28	33.35±13.67	31.87±13.40	<0.0001
Mean pack-years of smoking (±SD)	8,519	33.19±28.99	30.38±28.45	29.17±26.07	28.00±25.41	<0.0001
Alcohol consumption (%)^b^						
Non-drinkers	21,577	5,389 (81.22)	5,390 (81.24)	5,399 (81.37)	5,399 (81.37)	0.0003
1 to <7 drinks/week	3,772	904 (13.62)	918 (13.84)	960 (14.47)	990 (14.92)	
≥7 drinks/week	1,190	342 (5.15)	327 (4.93)	276 (4.16)	246 (3.71)	
Diabetes (%)^b^						
No	22,791	5,627 (84.81)	5,701 (85.92)	5,718 (86.18)	5,745 (86.59)	0.02
Yes	3,749	1,008 (15.19)	934 (14.08)	917 (13.82)	890 (13.41)	
Any weekly physical activity (%)^b^						
No	17,052	4,177 (62.95)	4,263 (64.25)	4,327 (65.21)	4,295 (64.73)	0.04
Yes	9,478	2,458 (37.05)	2,372 (35.75)	2,308 (34.79)	2,340 (35.27)	
Mean total energy intake (±SD), Kcal	26,540	1590.30±558.44	1598.18±565.38	1580.79±544.44	1597.44±572.57	0.25

*Relative telomere length was determined as the ratio of telomere repeat copy number (T) to single (albumin) gene copy number (S) in experimental samples relative to a reference sample

^a^Variables reported at baseline

^b^Variables reported at the follow-up 1.

Telomere length was positively associated with pancreatic cancer risk after adjustment for potential confounders (**Table 3**). Compared with the lowest quartile, participants with the highest quartile of telomere length were more than two times more likely to develop pancreatic cancer (HR = 2.18, 95% CI: 1.25–3.80, *P*_trend_ = 0.02). In the analysis of pancreatic adenocarcinoma only (n = 65), the results were comparable to the results that included all pancreatic cancer cases. The HRs (95% CIs) were 3.08 (1.17–8.11) for adenocarcinoma patients as compared with 2.29 (1.07–4.92) for all cases **(Table 3**). Although the association between telomere length and risk of non-adenocarcinoma or non-histologically confirmed pancreatic cancer was not linear and not statistically significant, the significantly elevated HR was observed for second quartile of telomere length (HR = 2.30, 95% CI: 1.06–4.96). The small sample size and heterogeneity of pancreatic cancer histology may contribute to the observed variation of risk estimates in this subgroup analysis. The difference in the telomere length-risk associations between the two histological subgroups was not statistically significant (*P*_*heterogeneity*_ = 0.308). Thus, all subsequent subgroup analyses were performed all pancreatic cancer cases.

**Table 3:** Association between relative average telomere length and pancreatic cancer risk in the Singapore Chinese Health Study, 1993–2016.

Relative average telomere length by pancreatic histology			
	Person-years	Number of cases	HR (95% CI)*
**Overall cases**			
Continuous Variable	348,979	116	2.29 (1.07–4.92)
Quartile Variable			
Q1 (shortest)	83,015	22	Ref.
Q2	86,507	35	1.81 (1.06–3.10)
Q3	88,240	25	1.45 (0.81–2.58)
Q4 (longest)	91,217	34	2.18 (1.25–3.80)
*P*_*trend*_			0.02
** Adenocarcinoma**			
Continuous Variable	348,549	65	3.08 (1.17–8.11)
Quartile Variable			
Q1 (shortest)	82,942	12	1.00
Q2	86,340	16	1.45 (0.68–3.07)
Q3	88,146	13	1.26 (0.57–2.80)
Q4 (longest)	91,121	24	2.50 (1.22–5.13)
*P*_*trend*_			0.02
**Non-Adenocarcinoma/unknown Histology**^**±**^			
Continuous Variable	348,424	51	1.47 (0.43–5.06)
Quartile Variable			
Q1 (shortest)	82,887	10	1.00
Q2	86,383	19	2.30 (1.06–4.96)
Q3	88,129	12	1.69 (0.72–3.96)
Q4 (longest)	91,025	10	1.63 (0.66–4.03)
*P*_*trend*_			0.23

^*^Adjusted for age, sex, education, dialect group, smoking status, alcohol drinking, BMI, diabetes history, and weekly physical activity.

^**±**^Including 38 pancreatic cancer cases with unknown histology.

In stratified analysis (**Table 4**), statistically significant association between longer telomeres and higher risk of pancreatic cancer was present in never smokers (HR = 3.04, 95% CI: 1.43–6.47 comparing the highest with the lowest quartile, *P*_trend_ = 0.007) but not in ever smokers, in men (HR = 2.91, 95% CI: 1.32–6.41, *P*_*trend*_ = 0.03) but not in women, in individuals without a history of diabetes (HR = 2.50, 95% CI: 1.33–4.70, *P*_*trend*_ = 0.01) but not in those with diabetes, or in those with <25 kg/m^2^ of BMI (HR = 2.44, 95% CI: 1.25–4.76 *P*_*trend*_ = 0.02) but not in their heavier counterparts. However, the heterogeneity in the telomere length-pancreatic cancer risk associations between these contrasting groups was not statistically significant (all *P*_*interaction*_ >0.05) (**Table 4**). When data were analyzed by the duration from blood collection to diagnosis, the association between telomere length and risk of pancreatic cancer was slightly stronger for shorter duration (<8 years) than that for longer duration (≥8 years); the corresponding HRs (95% CIs) of pancreatic cancer for the highest related to the lowest quartile were 3.11 (95% CI: 1.34–7.21) and 1.63 (95% CI: 0.78–3.41), respectively (**S1 Table**). However, their difference was not statistically significant (*P*_*heterogeneity*_ = 0.730). We repeated analysis after excluding first 3 years of data (i.e., both cases and person-years), the results remained the same (Data not shown).

**Table 4:** Association between relative average telomere length and pancreatic cancer risk among participants stratified by selected characteristics in the Singapore Chinese Health Study, 1993–2016.

Relative telomere length	Person-years	Number of cases	HR (95% CI)*
**By Smoking Status**			
Never Smokers			
Q1 (shortest)	52,980	10	Ref.
Q2	59,579	20	2.13 (0.99–4.56)
Q3	64,130	17	1.91 (0.87–4.21)
Q4 (longest)	69,061	25	3.04 (1.43–6.47)
*P*_*trend*_			0.007
Ever Smokers			
Q1 (shortest)	30,035	12	Ref.
Q2	26,928	15	1.54 (0.72–3.32)
Q3	24,110	8	1.02 (0.41–2.52)
Q4 (longest)	22,155	9	1.35 (0.56–3.28)
*P*_*trend*_			0.71
*P*_*interaction*_			0.23
**By Gender**			
Male			
Q1 (shortest)	43,313	11	Ref.
Q2	40,081	22	2.58 (1.25–5.34)
Q3	36,365	11	1.59 (0.68–3.70)
Q4 (longest)	33,296	16	2.91 (1.32–6.41)
*P*_*trend*_			0.03
Female			
Q1 (shortest)	39,702	11	Ref.
Q2	46,426	13	1.15 (0.51–2.57)
Q3	51,875	14	1.24 (0.56–2.76)
Q4 (longest)	57,920	18	1.57 (0.72–3.40)
*P*_*trend*_			0.24
*P*_*interaction*_			0.64
**By Diabetes History**			
No Diabetes History			
Q1 (shortest)	71,672	16	Ref.
Q2	75,455	29	2.05 (1.11–3.78)
Q3	77,296	21	1.64 (0.85–3.18)
Q4 (longest)	79,890	29	2.50 (1.33–4.70)
*P*_*trend*_			0.01
Diabetes History			
Q1 (shortest)	11,343	6	Ref.
Q2	11,052	6	1.17 (0.38–3.65)
Q3	10,944	4	0.92 (0.26–3.30)
Q4 (longest)	11,327	5	1.35 (0.40–4.58)
*P*_*trend*_			0.74
*P*_*interaction*_			0.28
**By BMI Satus**			
BMI<25			
Q1 (shortest)	60,735	15	Ref.
Q2	62,344	24	1.89 (0.99–3.62)
Q3	63,941	17	1.55 (0.77–3.13)
Q4 (longest)	66,141	24	2.44 (1.25–4.76)
*P*_*trend*_			0.02
BMI≥25			
Q1 (shortest)	22,280	7	Ref.
Q2	24,163	11	1.64 (0.63–4.25)
Q3	24,300	8	1.24 (0.44–3.45)
Q4 (longest)	25,075	10	1.68 (0.62–4.55)
*P*_*trend*_			0.44
*P*_*interaction*_			0.78

^*^Adjusted for age, sex, education, dialect group, smoking status, alcohol drinking, BMI, diabetes history, and weekly physical activity, if applicable.

## Discussion

In the present analysis of the population-based prospective cohort study of 26,540 individuals with an average follow-up of 13 years, we found that that longer telomeres in peripheral blood leukocytes were associated with significant increase in the risk of pancreatic cancer. The duration of follow-up did not have significant impact on the association between telomere length and risk of pancreatic cancer.

Previous epidemiological studies [12–16] reported notably inconsistent results that both short [12–14] and long leukocyte [12,14–16] telomere lengths were associated with increased risk of pancreatic cancer. Two studies [12,14] reported a U-shaped association between telomere length and pancreatic cancer risk, i.e., both shorter and longer telomeres were associated with higher risk of pancreatic cancer. Accordingly, in a retrospective case-control study of 499 pancreatic cancer cases and 963 controls matched with frequencies of age group, sex and residence in Mayo Clinic, Skinner et al. [12] found a U-shaped association between leukocyte telomere length and risk of pancreatic cancer. The other study was conducted by Zhang et al. [14], a nested case-control study of 900 pancreatic cancer cases and 900 matched controls within the Prevention Study of Adolescent Population in Liaoning Province, China, which found a similar U-shaped association. In addition, three nested case-control studies of pancreatic cancer in Western populations also reported mixed results. One study in the U.S [13] of 386 pancreatic cancer cases and 896 controls from 5 prospective cohort studies and matched by year birth, prospective cohort, smoking status, fasting status at blood collection, found that longer telomeres were associated with significantly lower risk of pancreatic cancer. The other two studies, both in Europe [i.e., a case-control study of 193 pancreatic cancer cases versus 660 controls from the Alpha-Tocopherol Beta-Carotene Cancer Prevention (ATBC) Study [15] and a case-control study of 331 matched case-control pairs from the European Prospective Investigation into Cancer and Nutrition (EPIC) Cohort [16]], found that longer telomeres were associated with statistically significant higher risk of pancreatic cancer. The results of these European studies [15,16] were comparable with the finding of our current study.

The inconsistency of the association between leukocyte telomere length and pancreatic cancer risk in different studies might be due to differences in study design (retrospective vs. prospective studies), study populations (Asian vs. European descendants), variation in the length of follow-up, and different prevalence rates of established risk factors (i.e., smoking, diabetes, physical activity or obesity), time between blood collection and the diagnosis date of pancreatic cancer, methods used to measure telomere length in different laboratories, as well as other unmeasured potential confounders[17]. For example, smoking [28,29], diabetes [30] and obesity [31] or physical activity [32,33] are known to be associated with telomere length as well in our study [26]. Lynch et al. [15] reported a positive association between telomere length and pancreatic cancer risk in the Alpha-Tocopherol Beta-Carotene Cancer Prevention Study that comprised male smokers only while in both Skinner et al. [12] and Zhang et al. [14] studies, the prevalence of current smokers at baseline for cases and controls were 11% vs. 32% and 31% vs. 20%, respectively; which were different from ours (i.e., 17.1% in cases vs. 16.2% in controls). Additionally, current participants of the Singapore Chinese Health Study were leaner (mean BMI = 23.3 in cases and 23.2 in non-cases) compared to study participants of all other five studies [12–16] where mean BMI was more than 25.0. The history of diabetes in our study (20% in cases and 14.1% in controls) was much lower than that in the studies by Skinner et al.[12] (74.5% in cases and 48.3% in controls) and Zhang et al. [14] (43.0% in cases and 39.3% in controls). Consequently, the differences in age and BMI, and proportions of male subjects, smokers and diabetics among various study populations could yield differential association between telomere length and pancreatic cancer risk.

In addition, the difference in time interval between dates of blood collection and diagnosis of pancreatic cancer might also contribute to the inconsistency of results from different studies. In the Alpha-Tocopherol Beta-Carotene Cancer Prevention (ATBC) Study, Lynch et al. [15] found that the positive association between telomere length and pancreatic cancer risk was observed in subjects diagnosed within the first five years of blood draw, but not those diagnosed greater than five years after blood draw. Conversely, in another study of five US prospective cohorts, Bao et al. [13] reported that shorter pre-diagnostic leucocyte telomere length was associated with increased risk of pancreatic cancer. In our study, we examined but did not find the duration of follow-up had significant impact on the risk of pancreatic cancer associated with telomere length. The different results by time intervals between dates of blood collection and diagnosis of pancreatic cancer (<8 years vs. ≥8 years) might be reflective of tumor adaptive mechanism for escaping cellular senescence and apoptosis. Telomere elongation at the initial stages of tumorigenesis may be due to activation of telomerase to ensure that tumor cells escape apoptosis and proceed to divide and proliferate indefinitely, suggesting a possible reverse causality [17], Our observed association that was statistically significant only within the first 8 years supports this speculation.

Studies using genetic variants as proxy measures for telomere length could avoid the potential biases due to disease progression and treatment, and other attributes to telomere shortening (i.e., aging and oxidative stress), reverse causation issue or inter-laboratory variability in the measurement of telomere length. Recently, Antwi et al. [18] constructed a genetic risk score of eight polymorphisms that were identified to be associated with leukocyte telomere length in genome-wide association study [34] and found no association between this genetic risk score and pancreatic cancer risk. Noted that this study only involved 1,500 cases and 1,499 non-cancer controls, a modest sample size for such type of effort. They, however, found that one short telomere-related allele (*rs10936599*, T) was associated with decreased risk of pancreatic cancer whereas another short telomere-related allele (*rs2736100*, A) was associated with increased risk of pancreatic cancer [18]. However, a recent study by Haycock et al. [35], using Mendelian randomization approach of GWAS data in a larger sample size (n = 5,105 pancreatic cancer cases versus 8,739 controls) found that the risk estimate for pancreatic cancer associated genotypes related to longer telomeres was not statistically significant (OR = 0.86, 95% CI: 0.57–1.32). Another approach might be to evaluate whether the association between leukocyte telomere length and pancreatic cancer risk is modulated by individual variation in telomere maintenance genes, such as *TERT*, *TRC*, *TRF1*, *TRF2*, *POT1* or *Rap1*. Indeed, in a nested case-control study of five cohorts, Bao et al. [13] found that three SNPs (*rs401681*, *rs2736100*, *rs2736098*) at *TERT* were associated with pancreatic cancer risk, of which the minor allele for *rs401681* was associated with shorter telomeres.

Several experimental studies have indicated that telomere length in peripheral blood leukocyte was positively correlated with telomeres in buccal cells [36], fibroblasts [36], skin [37,38], and synovial membrane [38]. A study by Daniali et al. [39] found that telomere length in peripheral blood leukocytes were highly correlated with those in the muscle cells (*r*^*2*^ = 0.71), fat (*r*^*2*^ = 0.69), and skin (*r*^*2*^ = 0.69) (all *P*s <0.0001). Furthermore, Gardner et al. [40] showed that telomere length in both skin and skeletal muscle cells was highly correlated with that in the pancreas tissue after controlling for chronological age. These data strongly suggest that peripheral blood leukocytes can be used as non-invasive surrogates for pancreas and other tissue types in the measurement of telomere length.

The biological mechanism linking longer telomere to pancreatic cancer is unclear. It is well known that in normal condition shorter telomeres may act as tumor suppressors [41] that can protect against carcinogenesis by triggering programmed cell death in the presence of functional cell cycle checkpoints and intact apoptotic pathways [42]. In contrast, cells with longer telomeres have higher proliferative capacity and more cell division. Each round of genome replication has the potential to introduce genetic mutations and chromosomal alterations, which may promote malignant transformation [43]. In addition, the mobilization of younger immune response cells, such as T cells is associated with longer telomeres [44,45], which might be involved in the promotion of carcinogenesis [46]. Furthermore, peripheral blood leukocyte telomere length may serve as an indicator of other factors for pancreatic cancer risk. Accordingly, smoking or oxidative stress might lead to telomere shortening, which then triggers cell mechanism such as increase in telomerase activity or activation of the telomerase independently, resulting in telomere lengthening [47]. Further studies are thus needed to elucidate the biological mechanisms for long telomeres in the development of pancreatic cancer.

Our study has several strengths. The prospective design minimized the potential impact of progression and treatment of pancreatic cancer on telomere length, since telomere length was determined on an average of 8.5 years before pancreatic cancer diagnosis. The long-term and complete follow-up further reduced potential bias due to the impact of undiagnosed pancreatic cancer on telomere length. A comprehensive adjustment for smoking, alcohol use, physical activity, BMI and history of diabetes minimized their potential confounding effect on the telomere length-pancreatic cancer risk association.

Our study also has some limitations. First, telomere length was measured in leukocyte rather than in target tissue. It is noted, however, that prior study shown high correlation of telomere length measures between the two tissue types [39,40]. Second, telomere length was measured at one-time point, which may not representative for true telomere length over time. This one-time point measurement would preclude our ability to evaluate the attrition rate of telomere length and the risk of pancreatic cancer development overtime. Third, relatively small number of cases hampered sub-group analyses, resulting in wide confidence intervals of risk estimates. Forth, as with any observational studies, residual confounding from measured (e.g., BMI, smoking and physical inactivity) or unmeasured factors could not be completely ruled out.

In summary, our study shows a dose-dependent association for peripheral blood telomere length with increased risk of pancreatic cancer. These findings, together with results from prior studies support a potential etiological role of longer telomeres for the development of pancreatic cancer. Future research efforts are warranted to elucidate the biological mechanism for longer telomeres in the development of pancreatic cancer.

## Supporting information

S1 Table Association between relative average telomere length and pancreatic cancer risk among participants stratified by median duration from blood collection to diagnosis in the Singapore Chinese Health Study, 1993–2016. (DOCX)
