# Supplementary material for: Association between leukocyte telomere length and the risk of pancreatic cancer: Findings from a prospective study
Source: PLoS One. 2019 Aug 29;14(8):e0221697. doi: 10.1371/journal.pone.0221697 (PMC6715276; doi:10.1371/journal.pone.0221697)
Supplement: S1 Table — (DOCX) [file pone.0221697.s001.docx]

**S1 Table. Association Between Relative Average Telomere Length and Pancreatic Cancer Risk among Participants Stratified by Median Duration from Blood Collection to Diagnosis in the Singapore Chinese Health Study, 1993-2016**

| **Relative telomere length** | **Person-years** | **Number of cases** | **HR^a^ (95% CI)^*^** |
| --- | --- | --- | --- |
|  |  |  |  |
| <8 years |  |  |  |
| Q1 (shortest) | 49,525 | 9 | Ref. |
| Q2 | 50,574 | 16 | 2.20 (0.97-4.99) |
| Q3 | 50,934 | 11 | 1.79 (0.73-4.37) |
| Q4 (longest) | 51,454 | 16 | 3.11 (1.34-7.21) |
| *P_trend_* |  |  | 0.02 |
| ≥8 years |  |  |  |
| Q1 (shortest) | 33,490 | 13 | Ref. |
| Q2 | 35,932 | 19 | 1.55 (0.76-3.15) |
| Q3 | 37,328 | 14 | 1.22 (0.57-2.62) |
| Q4 (longest) | 39,763 | 18 | 1.63 (0.78-3.41) |
| *P_trend_* |  |  | 0.31 |

^*^Adjusted for age, sex, education, dialect group, smoking status, alcohol drinking, BMI, diabetes history, and weekly physical activity.
